# Greater variability in lipid measurements associated with kidney diseases in patients with type 2 diabetes mellitus in a 10-year diabetes cohort study

**DOI:** 10.1038/s41598-021-87067-4

**Published:** 2021-04-13

**Authors:** Eric Yuk Fai Wan, Esther Yee Tak Yu, Weng Yee Chin, Christie Sze Ting Lau, Anna Hoi Ying Mok, Yuan Wang, Ian Chi Kei Wong, Esther Wai Yin Chan, Cindy Lo Kuen Lam

**Affiliations:** 1 Department of Family Medicine and Primary Care, The University of Hong Kong, 3/F Ap Lei Chau Clinic, 161 Main Street, Ap Lei Chau, Hong Kong; 2 Department of Pharmacology and Pharmacy, The University of Hong Kong, Ap Lei Chau, Hong Kong; 3 Research Department of Practice and Policy, School of Pharmacy, University College London, London, UK; 4 Department of Pharmacology and Pharmacy, Centre for Safe Medication Practice and Research, The University of Hong Kong, Ap Lei Chau, Hong Kong; 5 Laboratory of Data Discovery for Health (D24H), Hong Kong Science and Technology Park, Sha Tin, Hong Kong

**Keywords:** Nephrology, Risk factors, Diseases, Kidney diseases

## Abstract

This study aimed to evaluate the associations between variability of lipid parameters and the risk of kidney disease in patients with type 2 diabetes mellitus. Low-density lipoprotein-cholesterol, total cholesterol to high-density lipoprotein-cholesterol ratio and triglyceride were specifically addressed in this study. This retrospective cohort study included 105,552 patients aged 45–84 with type 2 diabetes mellitus and normal kidney function who were managed under Hong Kong public primary care clinics during 2008–2012. Those with kidney disease (estimated glomerular filtration rate < 60 mL/min/1.73 m^2^ or urine albumin to creatinine ratio ≥ 3 mg/mmol) were excluded. Variabilities of low-density lipoprotein-cholesterol, total cholesterol to high-density lipoprotein-cholesterol ratio and triglyceride were determined using the standard deviation of the respective parameter obtained from a mixed effects model to minimize regression dilution bias. The associations between lipid variability and renal outcomes including incident kidney disease, renal function decline defined as ≥ 30% reduction in estimated glomerular filtration rate since baseline, and end-stage renal disease (estimated glomerular filtration rate < 15 mL/min/1.73 m^2^) were evaluated by multivariable Cox regression. After a median follow-up of 66.5 months (0.5 million person-years in total), 49,653 kidney disease, 29,358 renal function decline, and 1765 end-stage renal disease cases were recorded. Positive linear associations between low-density lipoprotein-cholesterol and total cholesterol to high-density lipoprotein-cholesterol ratio variabilities and the risk of all renal outcomes were demonstrated. However, no association between triglyceride variability and any outcome was found. Each mmol/L increase in low-density lipoprotein-cholesterol variability was associated with 20% (Hazard ratio 1.20 [95% CI 1.15–1.25]), 38% (Hazard ratio 1.37 [95% CI 1.30–1.45]), and 108% (Hazard ratio 2.08 [95% CI 1.74–2.50]) higher risk in incident kidney disease, renal function decline and end-stage renal disease respectively. Similarly, each unit increase in total cholesterol to high-density lipoprotein-cholesterol ratio variability was associated with 35% (Hazard ratio 1.15 [95% CI 1.10–1.20]), 33% (Hazard ratio 1.33 [95% CI 1.26–1.40]), and 75% (Hazard ratio 1.75 [95% CI 1.46–2.09]) heightened risk in incident kidney disease, renal function decline and end-stage renal disease respectively. Cholesterol variability may potentially be a useful predictor of kidney diseases in patients with type 2 diabetes mellitus. Attention should be drawn to cholesterol variability when managing diabetic patients and further research is warranted to investigate the modifiable risk factors for lipid variability.

## Introduction

Kidney diseases are invariably associated with a myriad of significant morbidities and mortalities. In the United States, kidney disease and end stage renal disease (ESRD) cost 64 billion and 34 billion USD, respectively, thereby constituting a predominant worldwide public healthcare burden^1^. With increasing evidence showing associations between lipids and kidney diseases in diabetic populations^2^, optimal lipid level targets for diabetes management have been raised by various international guidelines, in efforts to prevent the escalating prevalence of kidney disease and related mortalities^3,4^. Though the evidence has shown effects of intra-individual lipid variabilities on cardiovascular diseases (CVD)^5–8^, its associations with kidney disease in the diabetic population remains briefly addressed in the literature.

The impact of lipid variability on kidney disease, specific to a diabetic population, has been insufficiently explored. Four studies to date have investigated the associations between lipid variability and progression of renal disease^9–12^, and the effects of respective lipid parameters have been inconsistent. An Italian study provided evidence for heightened risk in renal decline with increased variability in both low-density lipoprotein-cholesterol (LDL-C) and high-density lipoprotein-cholesterol (HDL-C)^10^; though this was not replicated in another Taiwanese study, which solely demonstrated aggravating effects of HDL-C variability on diabetic nephropathy progression^9^. Additionally, although such studies have illuminated potential associations between renal disease and variabilities in lipid traits, most had relatively short follow-up periods, a limited small cohort, or included patients with baseline macroalbuminuria, with only two of the four studies evaluating kidney disease^9,10^. The inclusion of post-baseline measurements in the determination of cholesterol variability was also unclear^9,10,12^, and as a result potentially introducing informative censoring and immortal time bias^13,14^. Moreover, although two studies have focused on the detrimental effects of variability in LDL-C, HDL-C, total cholesterol (TC) and triglyceride (TG), the impacts of variability in TC to HDL-C ratio have rarely been analysed. Taking into consideration the clinical implications of TC to HDL-C ratio in CVD prediction models, which in turn contributes to the onset of renal dysfunction and kidney disease, it arguably provides a more overarching indication for the treatment of respective lipid traits^15,16^. Furthermore, of the four studies, only two demonstrated impact of lipid variabilities in diabetic populations, whilst the others addressed hypertensive or general populations. Here, the specificity to a diabetic population is important, given that diabetic patients displayed a higher risk of kidney disease and ESRD^17^, thus rendering their results inapplicable^9,11^. This calls for further research to enable a clearer apprehension of the underlying relationship between cholesterol variability and the progression of kidney disease and mortality in diabetic population.

This study aims to investigate the associations between the variabilities of respective lipid parameters, LDL-C, TC to HDL-C ratio and triglyceride, and the risk of kidney disease, renal function decline and ESRD in type 2 diabetes mellitus (T2DM) patients without kidney disease. This will enable a timely identification of those at risk, better predictions and earlier implementation of appropriate preventative measures.

## Materials and methods

### Study design

This retrospective cohort study was conducted between 1 January 2008 and 31 December 2012 with data obtained from the database of the Hong Kong Hospital Authority (HA). Managing over 43 public-sector hospitals, 49 specialist outpatient clinics and 73 primary care clinics, the HA attends over 90% of patients in Hong Kong with chronic diseases^18^. The data used in this study, including mortality data, was anonymous data from electronic health record database. Hence, no informed consent was required. All methods in this study were conducted in accordance with the relevant guidelines and regulations. Patients aged 45–84 with clinically diagnosed type 2 DM and managed in primary care were included in this study. The diagnosis of type 2 DM was determined by the International Classification of Primary Care-2 (ICPC-2) code of T90. Lipid variability was determined by three or more lipid readings, obtained from annual assessment during the 2-year period on or before baseline. The timeline of lipid measurements and outcome ascertainment in this study is illustrated in Supplementary Fig. 1. The baseline for each patient was determined by the date of first doctor consultation in the clinic or date of the latest record of lipid measurements within the subject inclusion period. Each patient was followed until the outcome event, the last visit before 31 December 2017 or the date of death, whichever occurred first. To demonstrate the impact of lipid variability on renal function decline in DM patients, patients with kidney disease at baseline, defined as estimated glomerular filtration rate (eGFR) < 60 mL/min/1.73 m^2^ or urine albumin to creatinine ratio (ACR) ≥ 3 mg/mmol, or less than three lipid measurements were excluded from this study. The cut-off value for ACR corresponds to albuminuria category A2 or above as specified in the Kidney Disease: Improving Global Outcomes (KDIGO) guideline^19^.

### Outcome measures

The three outcomes included: (1) kidney disease, coded as 585.3–585.6 and 586.x in the International Classification of Diseases, Ninth Edition, Clinical Modification (ICD-9-CM), or defined as eGFR < 60 mL/min/1.73 m^2^ or new onset albuminuria, defined as ACR ≥ 3 mg/mmol; (2) renal function decline defined as ≥ 30% reduction in eGFR since baseline; (3) ESRD, coded as 585.5–585.6 in ICD-9-CM, or defined as eGFR < 15 mL/min/1.73 m^2^.

### Ethics approval

Ethics approval for this study was granted by the Institutional Review Boards (IRB) of the University of Hong Kong/Hospital Authority Hong Kong West Cluster. Anonymous data was extracted from the database in Hong Kong Hospital Authority, and thus the informed consent from all study subjects is waived by the IRB of the Hong Kong Hospital Authority.

### Lipid measurements

Lipid levels were obtained from blood samples after an overnight fast in each subject, following standardised and universal blood-taking protocol across all clinics and hospitals in the HA. Cholesterol and triglyceride levels were determined using Roche diagnostics with automatic biochemical analyzer (Cobas C6000 or equivalent). Direct LDL-C levels were measured, unless TG levels exceed 4.0 mmol/L, in which LDL-C levels would subsequently be calculated by the Friedewald equation^20^.

### Lipid variability measurements

For each patient, mixed effects model was applied to estimate the usual lipid level and variability, in which the intra-individual variability was used as the random effect. In the mixed effects model, difference between lipid levels amongst individuals was considered to obtain more accurate lipid variability, in turn reducing the regression dilution bias in the result. Based on JAGS Version 4.3.0 (http://mcmc-jags.sourceforge.net/) and the R2jags package in R Version 3.6 (https://www.r-project.org/)^21,22^, Markov Chain Monte Carlo (MCMC), a method from the Bayesian framework, was used to construct the mixed effects model. In the output of MCMC, posterior mean of the random intercept and residual standard deviation was used for the respective estimation of usual lipid levels and lipid variability measurements, represented by the mean and standard deviation of lipid level corrected with regression dilution bias, respectively. Further information regarding the statistical theories and algorithms could be found in the Supplementary document and previous literature^23,24^.

### Baseline characteristics

Baseline characteristics consisted of age, gender, duration of DM, smoking status, body mass index (BMI), systolic blood pressure (SBP), diastolic blood pressure (DBP), Haemoglobin A1c (HbA1c), eGFR^25^, urine ACR, the Charlson’s comorbidity index^26,27^, the use of anti-diabetic drug (e.g. insulin, metformin, sulphonylurea and others), the use of anti-hypertensive drug [e.g. angiotensin converting enzyme inhibitors or angiotensin receptor blockers (ACEI/ARB), β-blockers, calcium channel blockers (CCB), diuretics and others (hydralazine, methyldopa, and prazosin)], statins, and fibrates. All laboratory assays were conducted in accredited laboratories by the College of American Pathologists, the Hong Kong Accreditation Service, or the National Association of Testing Authorities, Australia.

### Data analysis

The missing data for baseline characteristics was deduced from multiple imputation. With the chained equation method, each missing value was imputed based on all covariates and outcomes for five times, hence generating five different datasets, which was applied in the same analysis. The results were further pooled in accordance with Rubin’s rule^28^.

The patients involved were divided into quintiles based on their lipid variabilities. Descriptive statistics for patient’s characteristics in each group were summarized. The incidence rate of kidney disease, renal function decline and ESRD were calculated for each group, with its 95% confidence interval (CI) based on Poisson distribution. Multivariable Cox proportional hazards regressions were adjusted to the patient’s characteristics and their usual lipids levels to evaluate the association between variability in lipids and the risk of an event. The CI of the hazard ratio was estimated using the floating absolute risk, without the requirement of reference group for reporting the standard error^29^. The scaled Schoenfeld residuals against time for covariates was used to assess the proportional hazards assumption. To inspect the existence of multi-collinearity, variance inflation factor was calculated. For this study, the results showed the fulfilment of proportional hazards assumption amongst all models with no significant multi-collinearity. The analyses were also repeated with two variability measurements, the coefficient of variation (CV) and variability independent of mean (VIM) instead of standard deviation (SD), to ensure robustness. Restricted cubic splines with three knots in Cox models were drawn to check the nonlinear pattern between lipid variability and the risk of outcomes. Additionally, three sensitivity analyses were performed in this study. Firstly, a complete case analysis was conducted. Secondly, to avoid the reverse causality, subjects with follow-up period of less than 1 year were excluded. Thirdly, the 24-month patient inclusion period was extended to 36 months.

Patients were divided into subgroups to explore the different relationships between cholesterol variability and outcomes for variant baseline characteristics, including gender (male; female), age (45–54, 55–64, 65–74, 75-84 years), smoking status (non-smoker, smoker), duration of DM (< 5, ≥ 5 years), BMI (< 25, ≥ 25 kg/m^2^), usual cholesterol level (LDL-C: < 2.6, 2.6–4.3, ≥ 4.3 mmol/L; TC-HDL-C ratio: < 3.5, 3.5–5, ≥ 5; triglyceride: < 1.8, 1.8–2.3, ≥ 2.3 mmol/L), baseline SBP (< 130, ≥ 130 mmHg), HbA1c (< 53 mmol/mol (< 7), ≥ 53 mmol/mol (≥ 7%)), eGFR (< 90, ≥ 90 mL/min/1.73 m^2^), Charlson’s Index (< 4, ≥ 4), use of anti-hypertensive drugs (no, yes), use of anti-diabetic drugs (no, yes), use of statins (no, yes), and use of fibrates (no, yes). To prevent multiple comparisons, the p-values were adjusted by Bonferroni correction.

Two-tailed tests with p-value significance level of 0.05 were applied in this study. The statistical analysis was executed with Stata Version 15.1 (https://www.stata.com/).

## Results

A total of 105,552 patients were included in this study after taking into account all inclusion and exclusion criteria. As shown in Supplementary Table 1, most of the baseline characteristics had a completion rate of above 99%, except for the duration of DM (94.9%), BMI (93.3%), and urine ACR (62.3%). The average number of lipid measurements taken from each patient was 3.1 (SD 0.5) and the mean age was 63.7 years (SD 9.5), with males accounting for 47.3% of the selected patients. Other baseline characteristics of each lipid variability group were summarised in Table 1. The mean values of LDL-C, TC to HDL-C ratio and triglyceride variability were 0.49 (SD 0.24), 0.57 (SD 0.29) and 0.44 (SD 0.41) respectively.

**Table 1:** Descriptive statistics for baseline characteristics among patients stratified by LDL-C, TC to HDL-C ratio and triglyceride variability.

	LDL-C variability (mmol/L)					Overall (N = 105,552)
	< 0.290 (N = 21,111)	0.291–0.378 (N = 21,110)	0.379–0.488 (N = 21,111)	0.489–0.681 (N = 21,110)	≥ 0.682 (N = 21,110)	
**Baseline characteristics**						
Male	51.0%	49.1%	47.7%	47.2%	41.4%	47.3%
Age, years	64.0 ± 9.6	63.8 ± 9.5	63.4 ± 9.4	63.2 ± 9.4	64.0 ± 9.3	63.7 ± 9.5
Current smoker	9.9%	9.7%	9.7%	10.1%	9.0%	9.7%
SBP, mmHg	133.4 ± 16.2	133.7 ± 16.3	133.8 ± 16.7	133.7 ± 16.8	133.7 ± 16.9	133.7 ± 16.6
DBP, mmHg	74.3 ± 9.7	74.6 ± 9.7	74.6 ± 9.7	74.8 ± 9.9	74.2 ± 9.8	74.5 ± 9.8
HbA1c, %	7.1 ± 1.1	7.2 ± 1.1	7.2 ± 1.2	7.2 ± 1.2	7.2 ± 1.2	7.2 ± 1.2
BMI, kg/m^2^	25.2 ± 3.8	25.3 ± 3.8	25.3 ± 3.8	25.3 ± 3.8	25.4 ± 3.7	25.3 ± 3.8
Duration of DM, year	8.6 ± 6.6	8.3 ± 6.5	8.0 ± 6.3	7.7 ± 6.3	7.4 ± 6.3	8.0 ± 6.4
eGFR, mL/min/1.73 m^2^	106.8 ± 23.6	107.0 ± 25.4	106.9 ± 23.4	107.1 ± 24.0	105.7 ± 24.3	106.7 ± 24.2
Urine ACR, mg/mmol	1.0 ± 0.7	1.1 ± 0.7	1.1 ± 0.7	1.1 ± 0.7	1.1 ± 0.7	1.1 ± 0.7
Charlson Index	3.1 ± 1.3	3.1 ± 1.3	3.1 ± 1.3	3.1 ± 1.3	3.2 ± 1.3	3.1 ± 1.3
Use of anti-diabetic drugs	90.1%	88.0%	87.3%	86.9%	85.6%	87.6%
Use of anti-hypertensive drugs	74.5%	74.3%	74.3%	75.0%	78.5%	75.3%
Use of statins	18.8%	19.1%	22.1%	38.5%	70.9%	33.9%
Use of fibrates	2.5%	2.9%	3.5%	4.8%	5.8%	3.9%
Number of LDL-C measurements	3.08 ± 0.37	3.08 ± 0.39	3.09 ± 0.42	3.11 ± 0.48	3.13 ± 0.53	3.10 ± 0.44
Usual LDL-C, mmol/L	2.59 ± 0.54	2.88 ± 0.57	3.03 ± 0.57	3.13 ± 0.56	3.25 ± 0.49	2.98 ± 0.59
LDL-C variability, mmol/L	0.24 ± 0.03	0.33 ± 0.03	0.43 ± 0.03	0.57 ± 0.06	0.88 ± 0.20	0.49 ± 0.24

	TC to HDL-C ratio variability					Overall (N = 105,552)
	< 0.339 (N = 21,111)	0.340–0.449 (N = 21,110)	0.450–0.577 (N = 21,111)	0.578–0.770 (N = 21,110)	≥ 0.771 (N = 21,110)	
**Baseline characteristics**						
Male	42.4%	45.9%	47.3%	49.0%	51.9%	47.3%
Age, years	64.5 ± 9.5	63.8 ± 9.4	63.4 ± 9.5	63.5 ± 9.4	63.2 ± 9.5	63.7 ± 9.5
Current smoker	6.7%	8.4%	9.3%	10.8%	13.3%	9.7%
SBP, mmHg	133.0 ± 16.6	133.6 ± 16.4	133.8 ± 16.4	133.9 ± 16.6	134.0 ± 16.8	133.7 ± 16.6
DBP, mmHg	73.3 ± 9.6	74.4 ± 9.7	74.8 ± 9.7	74.9 ± 9.8	75.1 ± 9.8	74.5 ± 9.7
HbA1c, %	7.1 ± 1.1	7.2 ± 1.1	7.2 ± 1.1	7.2 ± 1.2	7.2 ± 1.2	7.2 ± 1.2
BMI, kg/m^2^	24.4 ± 3.8	25.2 ± 3.8	25.5 ± 3.8	25.6 ± 3.7	25.8 ± 3.7	25.3 ± 3.8
Duration of DM, year	9.0 ± 6.8	8.3 ± 6.5	7.9 ± 6.3	7.7 ± 6.3	7.1 ± 6.0	8.0 ± 6.4
eGFR, mL/min/1.73 m^2^	108.2 ± 23.6	107.3 ± 23.3	107.0 ± 26.2	105.8 ± 23.2	105.1 ± 24.2	106.7 ± 24.2
Urine ACR, mg/mmol	1.0 ± 0.7	1.0 ± 0.7	1.1 ± 0.7	1.1 ± 0.7	1.1 ± 0.7	1.1 ± 0.7
Charlson Index	3.2 ± 1.3	3.1 ± 1.3	3.1 ± 1.3	3.1 ± 1.3	3.1 ± 1.4	3.1 ± 1.3
Use of anti-diabetic drugs	88.1%	87.7%	87.9%	87.2%	87.1%	87.6%
Use of anti-hypertensive drugs	70.8%	73.9%	75.6%	77.5%	78.7%	75.3%
Use of statins	22.0%	23.5%	28.9%	39.4%	55.4%	33.9%
Use of fibrates	1.2%	2.1%	3.2%	4.5%	8.6%	3.9%
Number of TC to HDL-C ratio measurements	3.09 ± 0.40	3.09 ± 0.40	3.11 ± 0.46	3.12 ± 0.49	3.20 ± 0.62	3.12 ± 0.48
Usual TC to HDL-C ratio	3.15 ± 0.57	3.81 ± 0.64	4.16 ± 0.70	4.46 ± 0.73	4.94 ± 0.74	4.10 ± 0.91
TC to HDL-C ratio variability	0.27 ± 0.05	0.39 ± 0.03	0.51 ± 0.04	0.66 ± 0.06	1.02 ± 0.27	0.57 ± 0.29

	Triglyceride variability (mmol/L)					Overall (N = 105,552)
	< 0.185 (N = 21,111)	0.186–0.268 (N = 21,110)	0.269–0.383 (N = 21,111)	0.384–0.595 (N = 21,110)	≥ 0.596 (N = 21,110)	
**Baseline characteristics**						
Male	53.2%	47.9%	44.8%	44.4%	46.1%	47.3%
Age, years	64.4 ± 9.5	64.5 ± 9.5	64.0 ± 9.4	63.5 ± 9.4	62.0 ± 9.3	63.7 ± 9.5
Current smoker	8.4%	9.1%	9.0%	10.1%	11.8%	9.7%
SBP, mmHg	132.1 ± 16.6	133.6 ± 16.7	134.0 ± 16.6	134.4 ± 16.5	134.2 ± 16.4	133.7 ± 16.6
DBP, mmHg	72.8 ± 9.5	74.0 ± 9.7	74.5 ± 9.7	75.2 ± 9.7	76.1 ± 9.8	74.5 ± 9.7
HbA1c, %	7.1 ± 1.1	7.1 ± 1.1	7.2 ± 1.1	7.2 ± 1.2	7.3 ± 1.2	7.2 ± 1.2
BMI, kg/m^2^	23.8 ± 3.6	25.0 ± 3.7	25.7 ± 3.8	25.9 ± 3.7	26.1 ± 3.7	25.3 ± 3.8
Duration of DM, year	9.4 ± 7.1	8.3 ± 6.5	7.8 ± 6.3	7.4 ± 6.0	7.0 ± 5.8	8.0 ± 6.4
eGFR, mL/min/1.73 m^2^	109.7 ± 23.7	106.7 ± 23.7	106.1 ± 23.5	105.6 ± 25.4	105.5 ± 24.2	106.7 ± 24.2
Urine ACR, mg/mmol	1.0 ± 0.7	1.0 ± 0.7	1.1 ± 0.7	1.1 ± 0.7	1.1 ± 0.7	1.1 ± 0.7
Charlson Index	3.2 ± 1.3	3.2 ± 1.3	3.2 ± 1.3	3.1 ± 1.3	3.0 ± 1.3	3.1 ± 1.3
Use of anti-diabetic drugs	87.7%	87.2%	87.4%	87.3%	88.3%	87.6%
Use of anti-hypertensive drugs	67.4%	74.3%	77.0%	78.7%	79.3%	75.3%
Use of statins	27.3%	33.7%	36.3%	37.1%	34.9%	33.9%
Use of fibrates	1.2%	1.7%	2.2%	2.9%	11.5%	3.9%
Number of triglyceride measurements	3.09 ± 0.40	3.10 ± 0.44	3.11 ± 0.46	3.13 ± 0.49	3.27 ± 0.70	3.14 ± 0.51
Usual triglyceride	0.79 ± 0.16	1.13 ± 0.14	1.42 ± 0.15	1.76 ± 0.16	2.31 ± 0.30	1.48 ± 0.56
Triglyceride variability	0.14 ± 0.03	0.22 ± 0.02	0.32 ± 0.03	0.47 ± 0.06	1.02 ± 0.58	0.44 ± 0.41

All parameters are expressed in either percentage or mean (standard deviation).

*BMI* Body mass index, *SBP* systolic blood pressure, *DBP* diastolic blood pressure, *HbA1c* haemoglobin A1c, *LDL-C* low-density lipoprotein-cholesterol, *TC* total cholesterol, *HDL-C* high-density lipoprotein-cholesterol, *eGFR *estimated glomerular filtration rate, *Urine ACR* urine albumin to creatinine ratio, *DM* diabeties mellitus.

**Figure 1:**
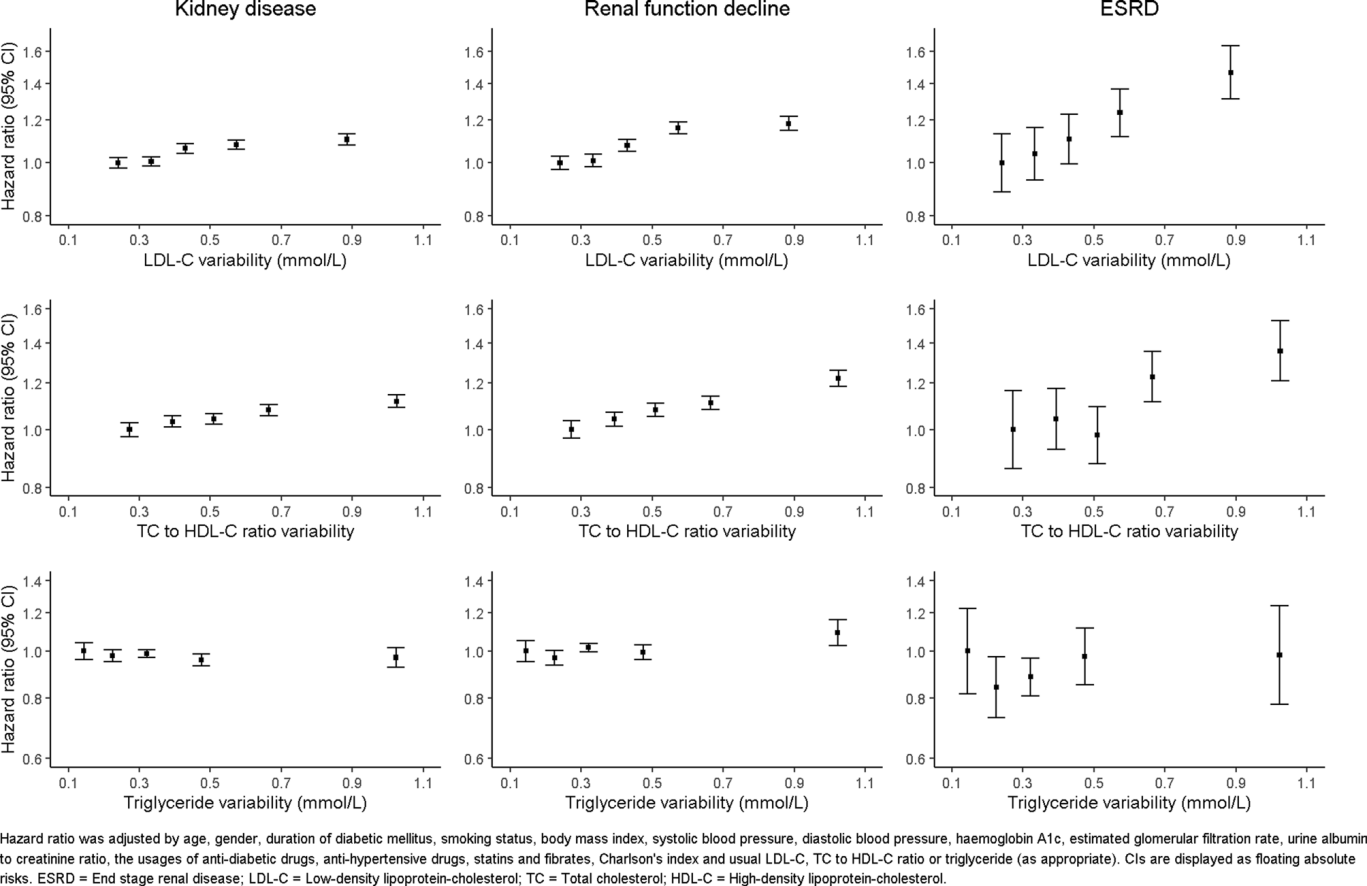
Hazard ratios for the association of a unit increase in LDL-C, TC to HDL-C ratio and triglyceride variability with kidney disease, renal function decline and ESRD from Cox regression models adjusted for baseline covariates.

After a median follow-up of 66.5 months (0.5 million person-years in total), 49,653 kidney disease, 29,358 renal function decline, and 1765 end-stage renal disease cases were recorded. Table 2 demonstrates the direct positive relationships between the variability of lipid traits LDL-C and TC to HDL-C ratio and incident rates of all outcomes. However, no association between TG variability and any renal outcome was found. Figure 1 also illustrates nearly identical patterns as above in the results of Cox regression adjusted with patient’s characteristics and usual lipid levels. In Supplementary Fig. 2a,b, similar trends were found between risk of outcomes and other variability measurements, including CV and VIM, compared with SD. Additionally, restricted cubic spline regression was utilized to test for non-linearity in the Cox models, as shown in Supplementary Fig. 3. Our findings demonstrated comparable patterns to the results above for the effect of lipid traits on the outcomes.

**Table 2:** Number, incidence rate and hazard ratio of kidney disease, renal function decline, and ESRD, stratified by LDL-C, TC to HDL-C ratio and triglyceride variability.

	LDL-C variability (mmol/L)					Overall (N = 105,552)
	< 0.290 (N = 21,111)	0.291–0.378 (N = 21,110)	0.379–0.488 (N = 21,111)	0.489–0.681 (N = 21,110)	≥ 0.682 (N = 21,110)	
**Kidney disease**						
Cumulative cases with event	9806	9771	9944	9985	10,147	49,653
Incidence rate (95% CI)^a^	91.3 (89.5, 93.2)	89.6 (87.8, 91.4)	92.2 (90.4, 94.0)	92.7 (90.9, 94.5)	97.1 (95.2, 99.0)	92.6 (91.7, 93.4)
Hazard ratio^b^ (95% CI)	1.00 (0.98, 1.02)	1.01 (0.99, 1.03)	1.06 (1.04, 1.08)	1.08 (1.06, 1.10)	1.10 (1.08, 1.13)	
**Renal function decline**						
Cumulative cases with event	5694	5700	5852	6087	6025	29,358
Incidence rate (95% CI)^a^	45.3 (44.2, 46.5)	44.8 (43.6, 45.9)	46.0 (44.9, 47.2)	48.5 (47.3, 49.7)	49.0 (47.8, 50.2)	46.7 (46.2, 47.3)
Hazard ratio^b^ (95% CI)	1.00 (0.97, 1.03)	1.01 (0.98, 1.04)	1.08 (1.05, 1.10)	1.16 (1.13, 1.19)	1.18 (1.15, 1.22)	
**ESRD**						
Cumulative cases with event	297	318	346	382	422	1765
Incidence rate (95% CI)^a^	2.1 (1.9, 2.4)	2.2 (2.0, 2.5)	2.4 (2.2, 2.7)	2.7 (2.4, 3.0)	3.0 (2.7, 3.3)	2.5 (2.4, 2.6)
Hazard ratio^b^ (95% CI)	1.00 (0.88, 1.13)	1.04 (0.93, 1.16)	1.11 (1.00, 1.23)	1.24 (1.12, 1.37)	1.46 (1.31, 1.64)	

	TC to HDL-C ratio variability					Overall (N = 105,552)
	< 0.339 (N = 21,111)	0.340–0.449 (N = 21,110)	0.450–0.577 (N = 21,111)	0.578–0.770 (N = 21,110)	≥ 0.771 (N = 21,110)	
**Kidney disease**						
Cumulative cases with event	9135	9688	9871	10,266	10,693	49,653
Incidence rate (95% CI)^a^	83.6 (81.9,85.3)	88.9 (87.1, 90.6)	91.5 (89.7, 93.3)	96.8 (95.0, 98.7)	102.6 (100.7, 104.6)	92.6 (91.7, 93.4)
Hazard ratio^b^ (95% CI)	1.00 (0.97, 1.03)	1.03 (1.01, 1.05)	1.04 (1.02, 1.06)	1.08 (1.06, 1.10)	1.12 (1.09, 1.14)	
**Renal function decline**						
Cumulative cases with event	5339	5637	5866	6020	496	29,358
Incidence rate (95% CI)^a^	42.4 (41.3, 43.6)	44.4 (43.3, 45.6)	46.4 (45.2, 47.6)	47.9 (46.7, 49.1)	52.6 (51.3, 53.8)	46.7 (46.2, 47.3)
Hazard ratio^b^ (95% CI)	1.00 (0.97, 1.03)	1.04 (1.01, 1.07)	1.08 (1.05, 1.11)	1.11 (1.08, 1.14)	1.22 (1.18, 1.26)	
**ESRD**						
Cumulative cases with event	266	306	305	407	481	1765
Incidence rate (95% CI)^a^	1.9 (1.7, 2.2)	2.2 (1.9, 2.4)	2.1 (1.9, 2.4)	2.9 (2.6, 3.1)	3.4 (3.1, 3.7)	2.5 (2.4, 2.6)
Hazard ratio^b^ (95% CI)	1.00 (0.86, 1.16)	1.04 (0.93, 1.17)	0.98 (0.88, 1.09)	1.23 (1.11, 1.35)	1.36 (1.21, 1.53)	

	Triglyceride variability (mmol/L)					Overall (N = 105,552)
	< 0.185 (N = 21,111)	0.186–0.268 (N = 21,110)	0.269–0.383 (N = 21,111)	0.384–0.595 (N = 21,110)	≥ 0.596 (N = 21,110)	
**Kidney disease**						
Cumulative cases with event	8476	9387	10,075	10,386	11,329	49,653
Incidence rate (95% CI)^a^	76.3 (74.7, 77.9)	86.3 (84.6, 88.1)	94.4 (92.6, 96.3)	97.8 (95.9, 99.7)	109.3 (107.3, 111.3)	92.6 (91.7, 93.4)
Hazard ratio^b^ (95% CI)	1.00 (0.96, 1.04)	0.98 (0.95, 1.00)	0.99 (0.97, 1.00)	0.96 (0.93, 0.99)	0.97 (0.93, 1.02)	
**Renal function decline**						
Cumulative cases with event	5310	5534	5933	5998	6583	29,358
Incidence rate (95% CI)^a^	42.7 (41.6, 43.9)	44.1 (42.9, 45.2)	47.3 (46.1, 48.5)	47.5 (46.3, 48.7)	51.9 (50.7, 53.2)	46.7 (46.2, 47.3)
Hazard ratio^b^ (95% CI)	1.00 (0.95, 1.05)	0.97 (0.94, 1.00)	1.02 (1.00, 1.04)	0.99 (0.96, 1.03)	1.09 (1.03, 1.16)	
**ESRD**						
Cumulative cases with event	287	287	330	394	467	1765
Incidence rate (95% CI)^a^	2.1 (1.9, 2.3)	2.1 (1.8, 2.3)	2.3 (2.1, 2.6)	2.8 (2.5, 3.0)	3.2 (2.9, 3.5)	2.5 (2.4, 2.6)
Hazard ratio^b^ (95% CI)	1.00 (0.82, 1.22)	0.84 (0.73, 0.97)	0.88 (0.81, 0.97)	0.98 (0.85, 1.12)	0.98 (0.78, 1.24)	

*ESRD* End stage renal disease, *LDL-C* low-density lipoprotein-cholesterol, *TC* total cholesterol, *HDL-C* high-density lipoprotein-cholesterol, *CI* confidence interval.

^a^Incidence rate (cases/1000 person-years) with 95% CI based on Poisson Distribution.

^b^Hazard ratio was adjusted by age, gender, duration of diabetic mellitus, smoking status, body mass index, systolic blood pressure, diastolic blood pressure, haemoglobin A1c, estimated glomerular filtration rate, urine albumin to creatinine ratio, the usages of anti-diabetic drugs, anti-hypertensive drugs, statins and fibrates, Charlson's index and usual LDL-C, TC to HDL-C ratio or triglyceride (as appropriate).

**Figure 2:**
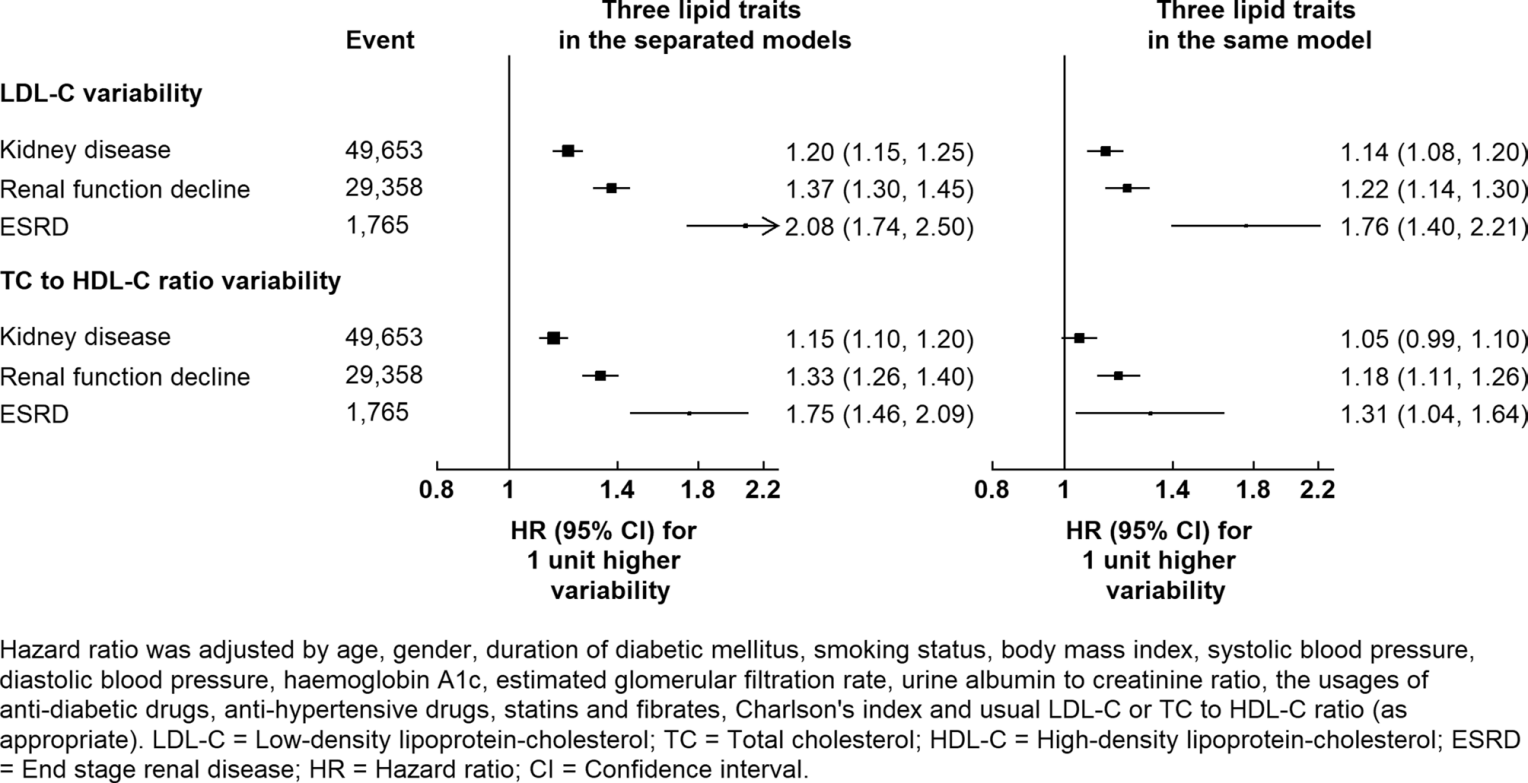
Hazard ratios for the risk of kidney disease, renal function decline and ESRD with each 1 unit increasing LDL-C or TC to HDL-C ratio variability using Cox regressions adjusted for baseline covariates.

Figure 2 exhibits significant associations between LDL-C and TC to HDL-C ratio variabilities and all three outcomes as these traits were associated with the outcomes suggested above. Each 1 mmol/L increase in LDL-C variability was associated with 20% (HR 1.20 [95% CI 1.15–1.25]), 38% (HR 1.37 [95% CI 1.30–1.45]), and 108% (HR 2.08 [95% CI 1.74–2.50]) higher risk in kidney disease, renal function decline and ESRD, respectively. Similarly, each 1 unit increase in TC to HDL-C ratio variability was found to be associated with 15% (HR 1.15 [95% CI 1.10–1.20]), 33% (HR 1.33 [95% CI 1.26–1.40]), and 75% (HR 1.75 [95% CI 1.46–2.09]) heightened risk in kidney disease, renal function decline and ESRD, respectively. The amalgamation of LDL-C and TC and HDL-C ratio variabilities in the same model demonstrated a significant yet slightly reduced effect, indicating that LDL-C and TC to HDL-C ratio variability were independently associated with renal dysfunction. To ensure robustness, three sensitivity analyses were conducted including: (1) Supplementary Fig. 4a which is complete case analysis, (2) Supplementary Fig. 4b which shows the result after excluding patients with follow-up period less than 1 year, and (3) Supplementary Fig. 4c which shows the result when patient inclusion period was extended from 24 to 36 months. These sensitivity analyses displayed similar results, which reaffirms the validity of the analysis. The result of further analysis on dividing the kidney disease into two group (i) eGFR < 60 mL/min/1.73 m^2^ and (ii) ACR ≥ 3 mg/mmol was shown in Supplementary Figs. 5 and 6. The LDL-C and TC to HDL-C ratio variability were associated with the outcomes except that TC to HDL-C ratio variability was marginal insignificant with the ACR ≥ 3 mg/mmol.

**Figure 3:**
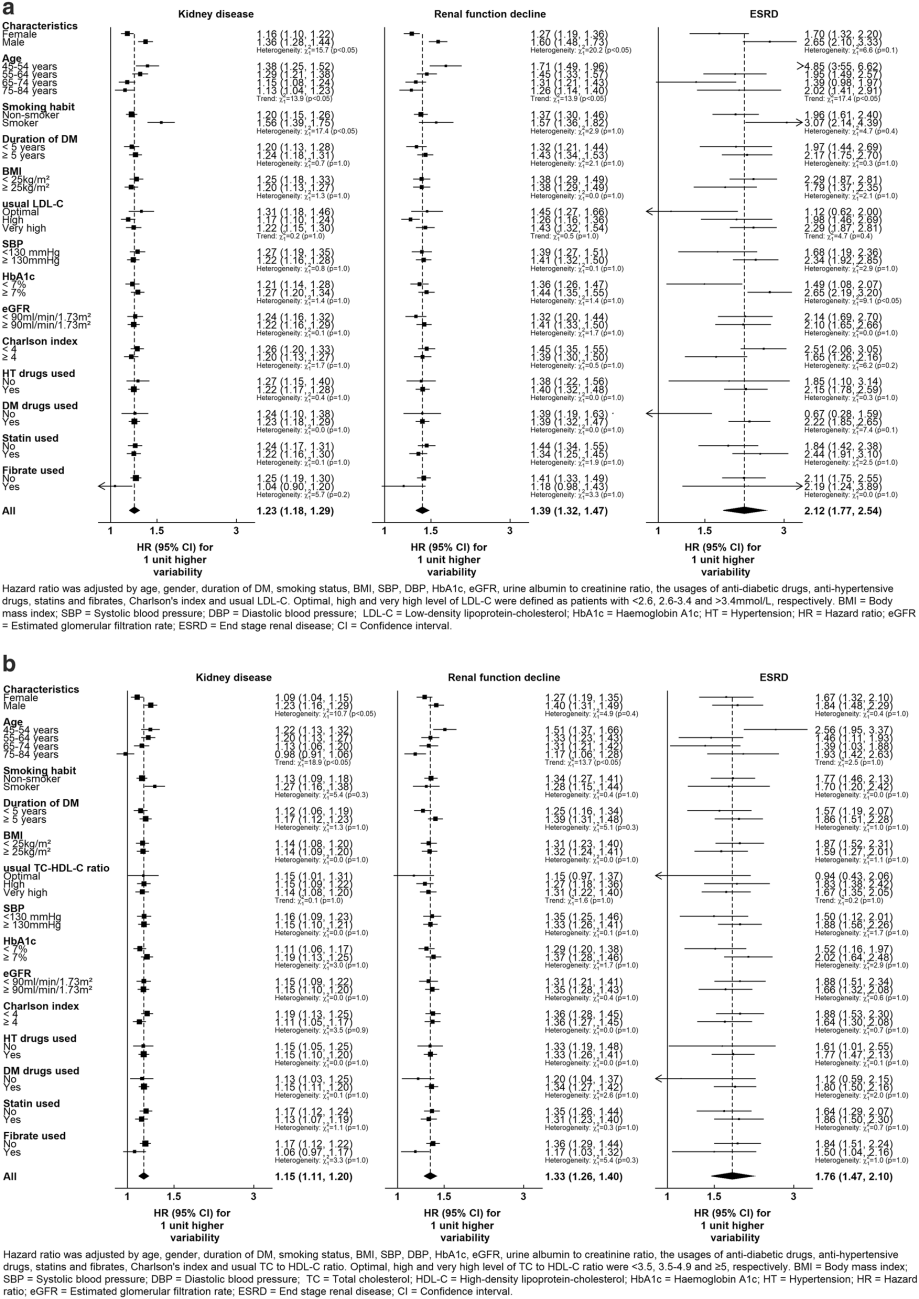
(**a**) Hazard ratios for the association of a unit increase in LDL-C variability with kidney disease, renal function decline and ESRD from Cox regression models adjusted for baseline covariates. (**b**) Hazard ratios for the association of a unit increase in TC to HDL-C ratio variability with kidney disease, renal function decline and ESRD from Cox regression models adjusted for baseline covariate.

Further results of subgroup analyses are denoted in Fig. 3a,b. Most baseline characteristics did not display an impact on the associations between lipid variability and renal dysfunction, apart from qualities such as age and gender. In particular, a negative association between age and the effect of lipid variability was observed, such that the risk of kidney disease in patients aged 45–54 years was approximately 22% higher than those aged 75–84 years, given the same degree in variability of LDL-C. Moreover, males demonstrated 10–50% higher LDL-C variability HR for the outcome events than females.

## Discussion

This is the first population-based study that demonstrated positive linear relationship between variabilities of LDL-C and TC to HDL-C ratio and the risk of kidney disease, renal function decline and ESRD in Chinese patients with T2DM. Nevertheless, triglyceride variability seemed to have an insignificant effect on any of the outcomes. Furthermore, patients of younger age were more susceptible to cholesterol variability in comparison to older patients, such that younger patients are at higher risk to kidney diseases despite same degree of variability in LDL-C and TC to HDL-C ratio in older patients. Therefore, cholesterol variability may be a potential indicator of diabetic nephropathy, and the importance of monitoring cholesterol variability in real-life practice should not be overlooked.

The effect of lipid variability on renal function decline was first discussed in a Taiwanese study, in which only HDL-C variability was found to be associated with a higher risk of diabetic nephropathy progression in patients with T2DM^9^. This could be attributed to their relatively small sample size and inclusion of patients with baseline macroalbuminuria. Furthermore, this study ties in well with an Italian study conducted in 2017, wherein the variability in HDL-C and LDL-C indicated the decline in eGFR in diabetic population^10^. However, the median of patients’ baseline eGFR was significantly lower than that of our study population, and they did not stratify the severity of renal function decline. In this study, variability of TC to HDL-C ratio was assessed in lieu of HDL-C variability, as TC to HDL-C ratio has shown to be a better indicator of target organ damage when compared to other lipid parameters^30^. Additionally, TC to HDL-C ratio played an important role in predicting cardiovascular risk, which has been included in the QRISK cardiovascular disease risk algorithm, with atherosclerosis as one of the major causes of renal failure^31^. In terms of triglyceride level, this study did not find any significant association between TG variability and any of the outcomes, which parallels the findings in previous studies^9,10^. In short, this study demonstrated a positive correlation between all three outcomes and the variability in LDL-C and TC to HDL ratio, but further research is still warranted as there has only been limited studies documenting the effect of LDL-C and TC to HDL-C ratio variability on renal function decline in diabetic population and future work could reaffirm the validity of cholesterol as the indicator of diabetic nephropathy.

Current literature has almost exclusively focused on the correlation between lipid variability and increased cardiovascular risk, but research in renal dysfunction remains limited. It has been hypothesized that higher LDL-C variability could disrupt cholesterol-dependent plaque^8^, impair endothelial function and inhibit lipid efflux from plaques^32^, thus increasing the risk of atherosclerosis. Similarly, these factors may also provide plausible explanations for the associations between LDL-C variability and renal function decline. It has been widely speculated that an increase in lipoprotein levels causes CVD and kidney disease with similar mechanisms, through heightened lipid trapping and adherence of monocytes to endothelial cells, thereby resulting in the development of focal glomerulosclerosis^33^. A post-hoc analysis provided evidence for the correlation between variability in TC to HDL-C ratio and percent atheroma volume progression^34^, supporting the atherosclerotic hypothesis in causing renal dysfunction. Furthermore, lipid variability could be an epiphenomenon of other conditions and frailty that increases risk of kidney disease^7,35^. Lastly, some studies suggested non-adherence to statins as one of the possible reasons^5,36^, though medication compliance was not addressed in the current study and the effect of lipid variability remained significant after adjusting for the use of lipid-lowering agents.

Patients of different age range and gender illustrated different outcomes. In this study, the effect of each unit increase in lipid variability on escalating kidney disease risk was more prominent in younger patients. This could be due to the fact that older patients are generally more vulnerable and have more comorbidities, hence masking the effect of lipid variability and in turn resulting in an age-specific difference. In addition, male patients demonstrated a higher risk in kidney disease when compared to females with the same unit increase in lipid variability, though previous studies provided evidence that females exhibit more variable LDL-C levels than males^5,6,8^, and further research is required to elucidate the exact underlying mechanism. Minor changes in levels of LDL-C, HDL-C and TC throughout menstrual cycles have also been observed^37^, providing potential explanations for the reduced susceptibility to renal dysfunction in females, with their innate exposure to greater fluctuating microenvironments.

### Strengths and limitations

With the large sample size and appropriate study design, this cohort study yielded results of minimal bias, limiting all informative observation of lipid measurements, immortal time bias and regression dilution bias, thereby ensuring reliability and validity of results.

However, although the study casts a new light on effects of lipid variability on kidney disease, it presents some apparent limitations. As a retrospective cohort study, the conclusion drawn suffers from the limitation of solely illustrating an associative relationship between lipid variability and kidney disease. This would require further investigation into the underlying mechanisms, in order to elucidate a causal relationship between the two. Nevertheless, multiple confounders were adjusted for in our analyses, and despite the possibility of residual confounding, the potentiality of reverse causation remains negligible with the exclusion of patients with baseline kidney disease. Besides, the results are in parallel with those acquired from the sensitivity analysis that only included patients with follow-up period of more than one year. Furthermore, serum urate level, which could be an independent risk factor for incident kidney disease, was not included in the current analysis. Additionally, although this study failed to take into consideration the various behavioural or habitual characteristics of patients, including levels of physical activity, dietary intake, and medication compliance; anthropometric and clinical parameters that were deemed relevant, such as BMI, HbA1c and blood pressure, have been included to account for patients’ disease severity and lifestyle. Lastly, with associations between cholesterol variability and elevated kidney disease risk ascribed to potential individual differences in diabetic patients of the cohort, the results therefore may not be extended to non-diabetic population.

## Conclusion

This population-based cohort study demonstrated a positive linear association between variability in LDL-C and TC to HDL-C ratio and kidney diseases in Chinese T2DM patients; shedding new light on the effects of cholesterol variability on kidney diseases, further reinforcing the validity of TC to HDL-C ratio as a clinical indicator of kidney diseases. These results not only confirmed the hypothesis that increased cholesterol variability aggravates the progression of kidney diseases, but also revealed a greater impact in younger, male patients. Such conclusions may reaffirm the validity of cholesterol variability as a useful predictor for kidney diseases outcomes in patients with T2DM, subsequently enabling diabetic patients to better envisage and prevent kidney diseases.

## Supplementary Information

Supplementary Information.

## Abbreviations

ACEI/ARB: Angiotensin converting enzyme inhibitors or angiotensin receptor blockers
BMI: Body mass index
CCB: Calcium channel blockers
CVD: Cardiovascular diseases
CV: Coefficient of variation
CI: Confidence interval
DBP: Diastolic blood pressure
ESRD: End stage renal disease
eGFR: Estimated glomerular filtration rate
HbA1c: Haemoglobin A1c
HDL-C: High-density lipoprotein-cholesterol
HA: Hospital Authority
ICD-9-CM: International Classification of Diseases, Ninth Edition, Clinical Modification
ICPC-2: International Classification of Primary Care-2
IRB: Institutional Review Boards
LDL-C: Low-density lipoprotein-cholesterol
MCMC: Markov Chain Monte Carlo
SD: Standard deviation
SBP: Systolic blood pressure
T2DM: Type 2 diabetes mellitus
TG: Triglyceride
VIM: Variability independent of mean
