# Supplementary material for: Greater variability in lipid measurements associated with kidney diseases in patients with type 2 diabetes mellitus in a 10-year diabetes cohort study
Source: Sci Rep. 2021 Apr 13;11:8047. doi: 10.1038/s41598-021-87067-4 (PMC8044222; doi:10.1038/s41598-021-87067-4)
Supplement: Supplementary file 1 — Supplementary Information. [file 41598_2021_87067_MOESM1_ESM.pdf]

Greater variability in lipid measurements associated with kidney diseases in patients with type 2 diabetes mellitus in a 10-year diabetes cohort study

Authors: Eric Yuk Fai Wan, PhD[1 2]*, Esther Yee Tak Yu, MBBS[1], Weng Yee Chin, MD[1], Christie Sze Ting Lau, BSc[1], Anna Hoi Ying Mok, MCP[1], Yuan Wang, MStat[1], Ian Chi Kei Wong, PhD[2 3], Esther Wai Yin Chan, PhD[4], Cindy Lo Kuen Lam, MD[1]

[1]Department of Family Medicine and Primary Care, The University of Hong Kong, Hong Kong

[2]Department of Pharmacology and Pharmacy, The University of Hong Kong, Hong Kong

[3]Research Department of Practice and Policy, School of Pharmacy, University College London, United Kingdom

[4]Centre for Safe Medication Practice and Research, Department of Pharmacology and Pharmacy, The University of Hong Kong, Hong Kong

Corresponding author:

Dr. Eric Yuk Fai Wan

Address: Department of Family Medicine and Primary Care, the University of Hong Kong, 3/F Ap Lei Chau Clinic, 161 Main Street, Ap Lei Chau, Hong Kong.

Tel. (852) 2552 4690

Fax. (852) 2814 7475

Email: yfwan@hku.hk

Electronic Supplementary Material

Estimating cholesterol variability using a linear mixed effects model

Given a dataset with N individuals and n_i cholesterol measurements from the ith individual, $i = 1, \dots, N$, let Y_{ij}, $j = 1, \dots, n_i$, be the jth measurement of individual i taken at measurement time t_{ij}.

Consider a standard linear mixed effects model

$$Y_{ij} = \beta^T X_{ij} + b_i^T Z_{ij} + \varepsilon_{ij}$$

where X_{ij} is a covariate vector for the fixed effects β and Z_{ij} is a covariate vector for the random effects b_i, assumed normally distributed $b_i \sim N(0, \Sigma_b)$. The residual errors ϵ_{ij} are assumed independent and normally distributed, $\epsilon_{ij} \sim N(0, \sigma^2)$. We can allow variability in the repeated measurements to differ between individuals by replacing the residual SD σ with an individual-specific residual SD σ_i and assuming that the σ_i are randomly distributed. We assume a log-normal distribution for the residual SD distribution, ensuring positivity of the SDs, $\sigma_i \sim \log N(\mu_\sigma, \tau_\sigma^2)$. The choice of log-normal distribution also allows a natural extension of the model to incorporate correlation between the usual level and the residual SD by assuming a multivariate normal distribution for the random effects and log residual SD

$$\varepsilon_{ij} \sim N(0, \sigma_i^2), \qquad \begin{pmatrix} b_i \\ \log \sigma_i \end{pmatrix} \sim N\left(\begin{pmatrix} 0 \\ \mu_\sigma \end{pmatrix}, \begin{pmatrix} \Sigma_b & \Sigma_{b\sigma} \\ \Sigma_{b\sigma}^T & \tau_\sigma^2 \end{pmatrix}\right)$$

where $\Sigma_{b\sigma}$ is a vector of covariances between the random effects and the random residual errors. For this study, the model was

$$\begin{pmatrix} b_i \\ \log \sigma_i \end{pmatrix} \sim N\left(\begin{pmatrix} 0 \\ \mu_\sigma \end{pmatrix}, \begin{pmatrix} \tau_0^2 & \rho\tau_0\tau_\sigma \\ \rho\tau_0\tau_\sigma & \tau_\sigma^2 \end{pmatrix}\right)$$

For the Bayesian estimation, we used diffuse uniform prior distributions U[0, 100] for SDs, uniform U[−1, 1] prior distributions for correlation parameters, and diffuse normal prior distributions $N(0, 100^2)$ for all other parameters. Priors were specified for the bivariate normal distribution by expressing it as two conditional univariate normal distributions. In the current study, we used a Markov Chain Monte Carlo (MCMC) of 2000 iterations (1000 burn-in) to update for the mixed effects models.

ESM Table 1. Data completion rate of the baseline characterstics in studied patients

	Total (N=105,552)
Age	100.0% (105,553)
Gender	100.0% (105,553)
Duration of diabetic mellitus	94.9% (100,189)
Smoking status	99.3% (104,770)
Body mass index	93.3% (98,482)
Systolic blood pressure	99.2% (104,736)
Diastolic blood pressure	99.2% (104,737)
Haemoglobin A1c	99.7% (105,206)
Low-density lipoprotein-cholesterol	100.0% (105,553)
Total cholesterol to high-density lipoprotein-cholesterol ratio	100.0% (105,553)
Triglyceride	100.0% (105,553)
Estimated glomerular filtration rate	100.0% (105,553)
Urine albumin to creatinine ratio	62.3% (65,723)
Charlson index	100.0% (105,553)
Use of anti-diabetic drugs	100.0% (105,553)
Use of anti-hypertensive drugs	100.0% (105,553)
Use of statins	100.0% (105,553)
Use of fibrates	100.0% (105,553)

ESM Figure 1. Study design for the investigation of the association between cholesterol variability and risk of kidney disease, renal function decline, and end-stage renal disease. The measurements of lipid between 2 years before baseline and baseline were used to calculate usual mean and variability of cholesterol. The median follow-up period was 78.5 months after baseline.

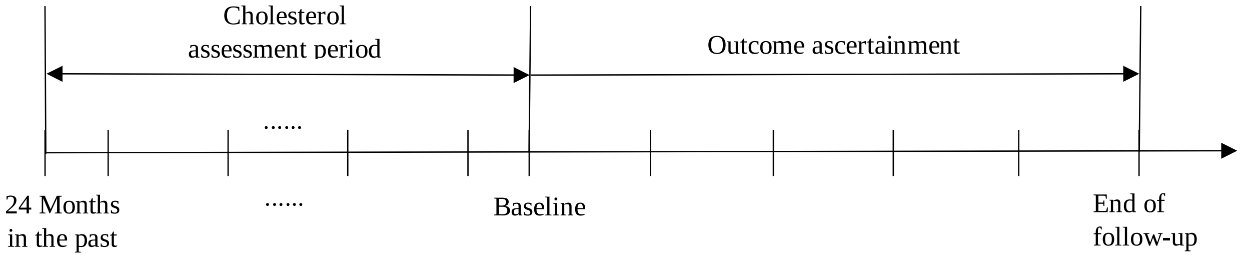

ESM Figure 2a. Hazard ratios for the association of a unit increase in LDL−C, TC to HDL−C ratio and triglyceride variability, measured using the coefficient of variation, with kidney disease, renal function decline and ESRD from Cox regression models adjusted for baseline covariates

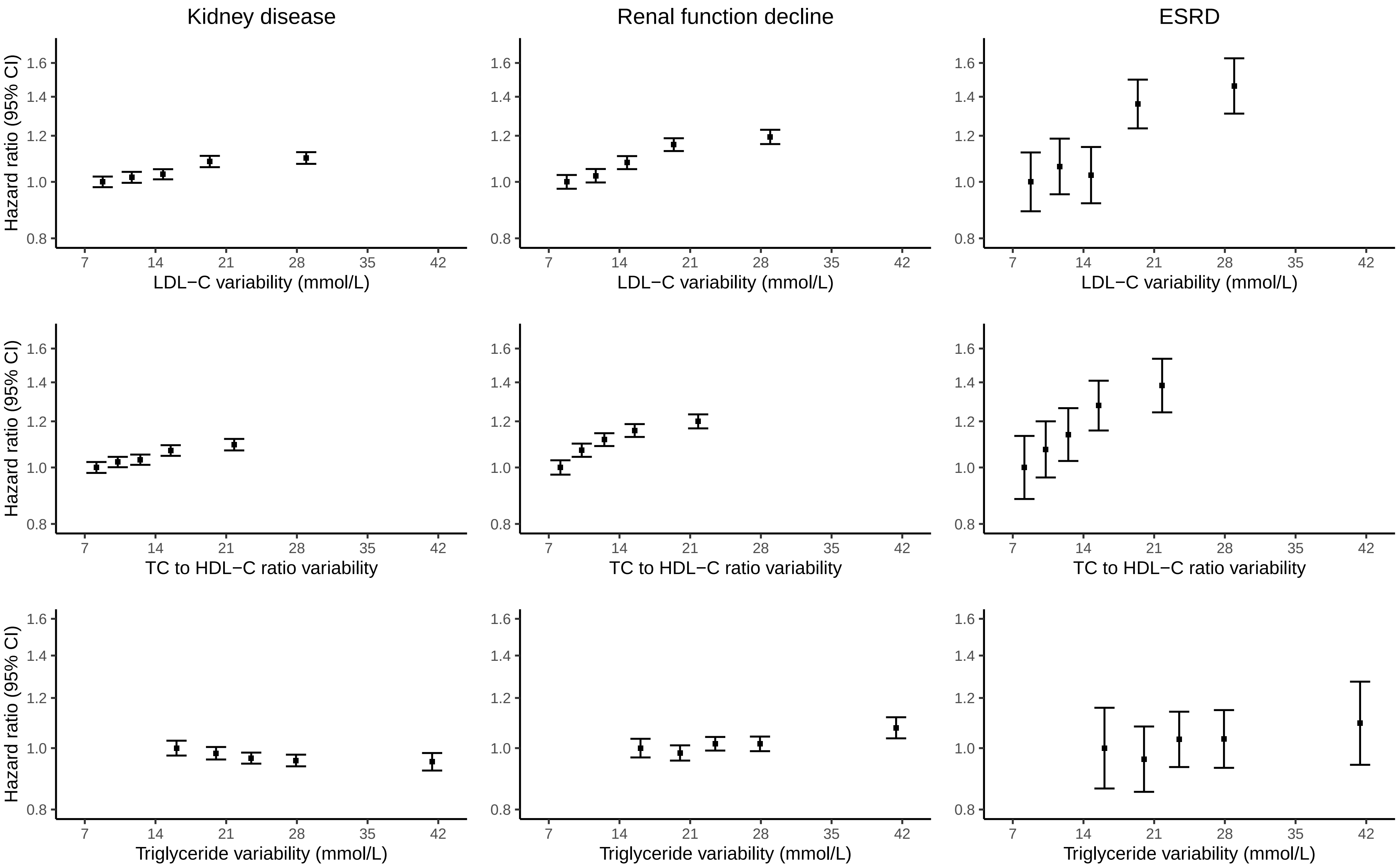

Hazard ratio was adjusted by age, gender, duration of diabetic mellitus, smoking status, body mass index, systolic blood pressure, diastolic blood pressure, haemoglobin A1c, estimated glomerular filtration rate, urine albumin to creatinine ratio, the usages of anti−diabetic drugs, anti−hypertensive drugs, statins and fibrates, Charlson's index and usual LDL−C, TC to HDL−C ratio or triglyceride (as appropriate). CIs are displayed as floating absolute risks. ESRD = End stage renal disease; LDL−C = Low−density lipoprotein−cholesterol; TC = Total cholesterol; HDL−C = High−density lipoprotein−cholesterol

ESM Figure 2b. Hazard ratios for the association of a unit increase in LDL−C, TC to HDL−C ratio and triglyceride variability, measured using the variability independent of the mean, with kidney disease, renal function decline and ESRD from Cox regression models adjusted for baseline covariates

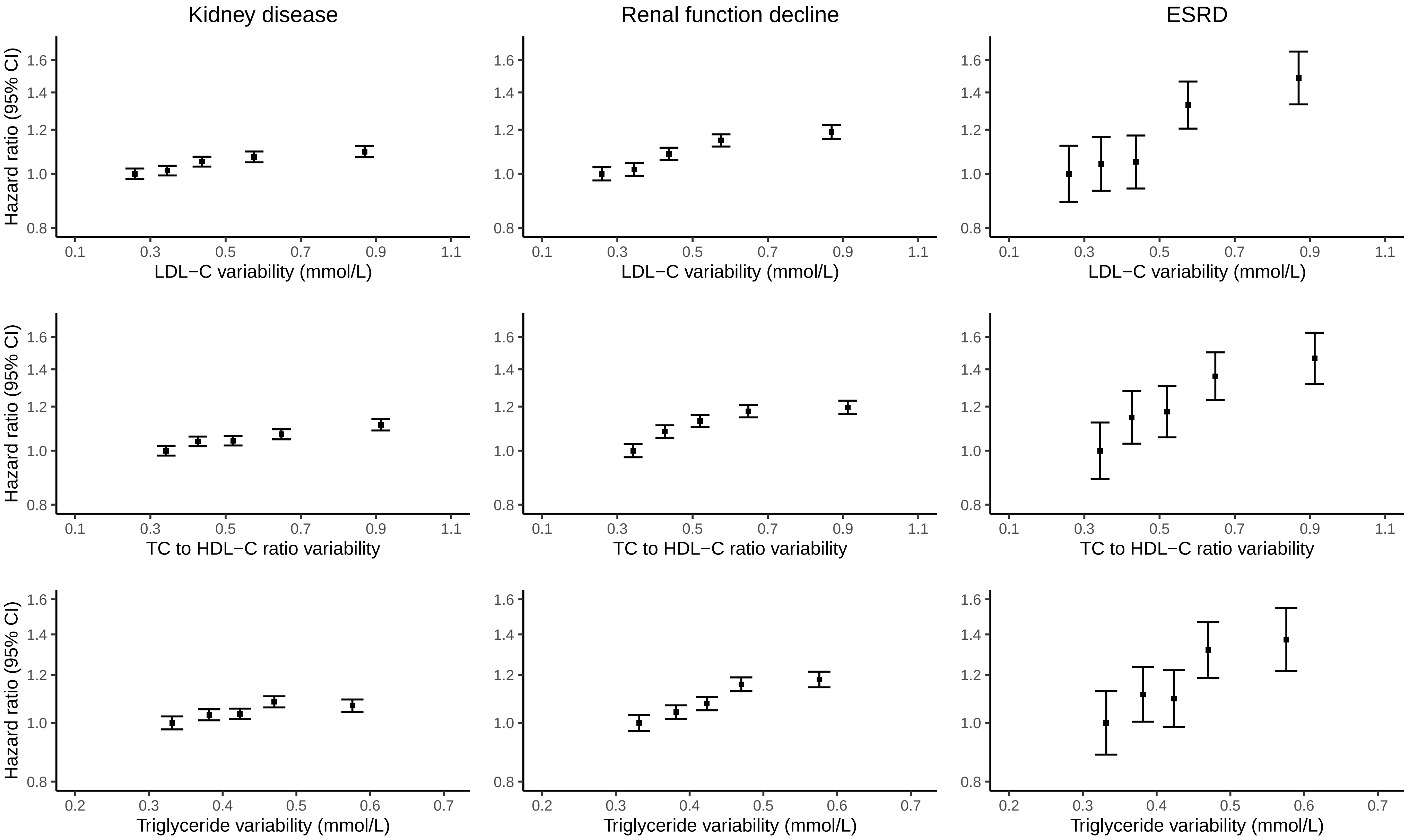

Hazard ratio was adjusted by age, gender, duration of diabetic mellitus, smoking status, body mass index, systolic blood pressure, diastolic blood pressure, haemoglobin A1c, estimated glomerular filtration rate, urine albumin to creatinine ratio, the usages of anti−diabetic drugs, anti−hypertensive drugs, statins and fibrates, Charlson's index and usual LDL−C, TC to HDL−C ratio or triglyceride (as appropriate). CIs are displayed as floating absolute risks. ESRD = End stage renal disease; LDL−C = Low−density lipoprotein−cholesterol; TC = Total cholesterol; HDL−C = High−density lipoprotein−cholesterol.

ESM Figure 3. Time-varying hazard ratios for the association of a unit increase in LDL-C, TC to HDL-C ratio and triglyceride variability with kidney disease, renal function decline and ESRD, estimated using restricted cubic splines in Cox regression models adjusted for baseline covariates

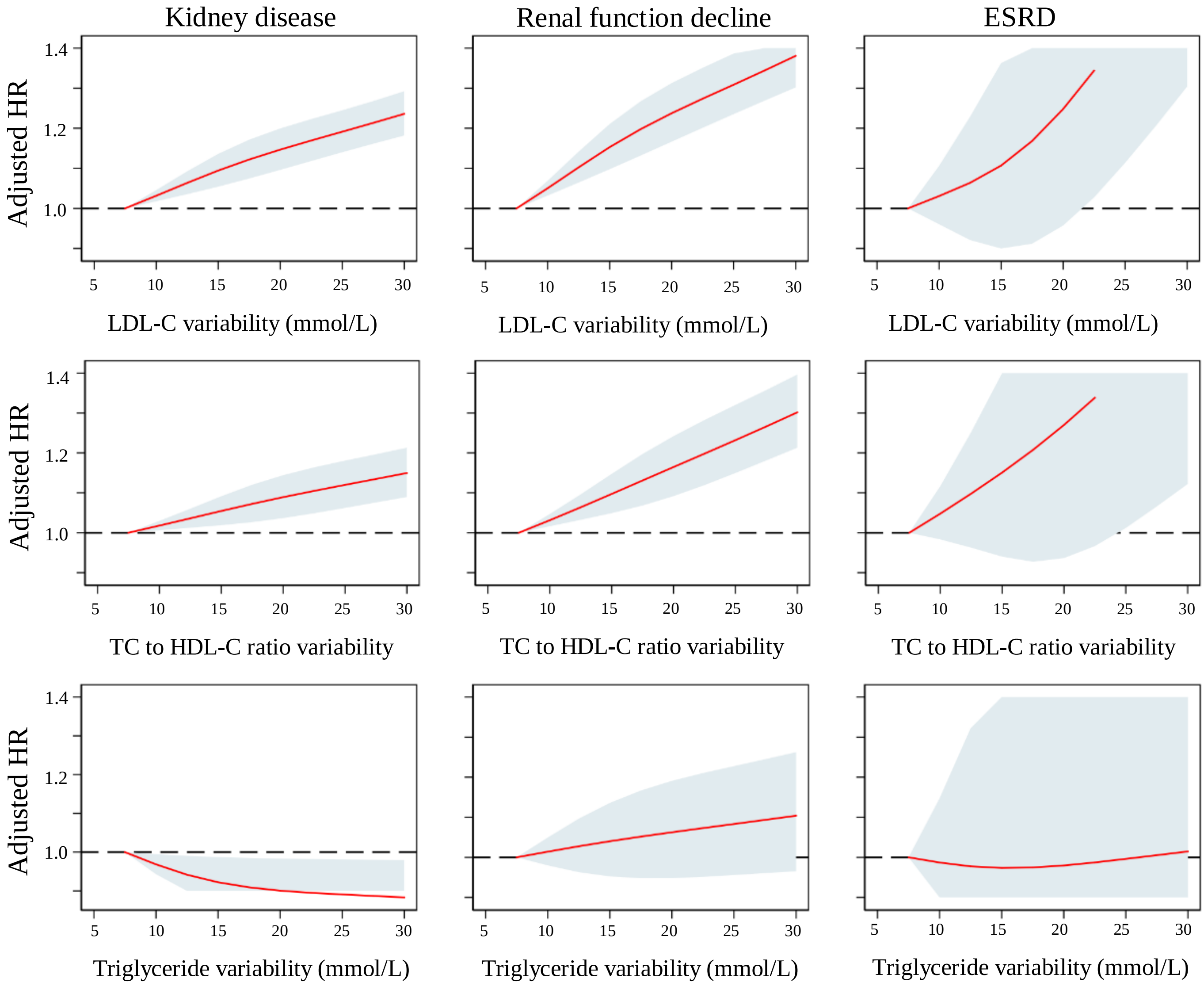

Hazard ratio was adjusted by age, gender, duration of diabetic mellitus, smoking status, body mass index, systolic blood pressure, diastolic blood pressure, haemoglobin A1c, estimated glomerular filtration rate, urine albumin to creatinine ratio, the usages of anti-diabetic drugs, anti-hypertensive drugs, statins and fibrates, Charlson's index and usual LDL-C, TC to HDL-C ratio or triglyceride (as appropriate). Shaded region represents 95% confidence intervals. ESRD = End stage renal disease; LDL-C = Low-density lipoprotein-cholesterol; HR = Harzard ratio.

ESM Figure 4a. Hazard ratios for the risk of kidney disease, renal function decline and ESRD with each 1 unit increase in LDL-C (mmol/L) or TC to HDL-C ratio variability using Cox regressions adjusted for baseline covariates in sensitivity analysis 1 (complete case analysis, i.e. exclude records with any missing value)

	Event	HR (95% CI)
**LDL-C variability**		
Kidney disease	27,330	1.17 (1.11, 1.24)
Renal function decline	14,282	1.25 (1.15, 1.35)
ESRD	529	1.64 (1.10, 2.44)
**TC to HDL-C ratio variability**		
Kidney disease	27,330	1.13 (1.06, 1.19)
Renal function decline	14,282	1.25 (1.16, 1.35)
ESRD	529	1.34 (0.90, 2.00)

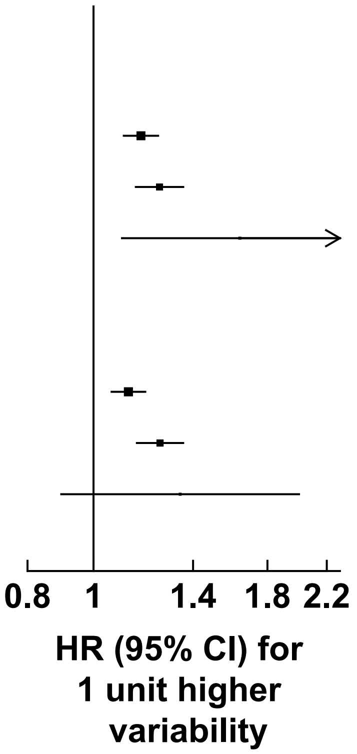

Hazard ratio was adjusted by age, gender, duration of diabetic mellitus, smoking status, body mass index, systolic blood pressure, diastolic blood pressure, haemoglobin A1c, estimated glomerular filtration rate, urine albumin to creatinine ratio, the usages of anti-diabetic drugs, anti-hypertensive drugs, statins and fibrates, Charlson's index and usual LDL-C or TC to HDL-C ratio (as appropriate). LDL-C = Low-density lipoprotein-cholesterol; TC = Total cholesterol; HDL-C = High-density lipoprotein-cholesterol; ESRD = End stage renal disease; HR = Hazard ratio; CI = Confidence interval.

ESM Figure 4b. Hazard ratios for the risk of kidney disease, renal function decline and ESRD with each 1 unit increase in LDL-C (mmol/L) or TC to HDL-C ratio variability using Cox regressions adjusted for baseline covariates in sensitivity analysis 2 (excluding patients with less than 12 months follow-up)

	Event	HR (95% CI)
**LDL-C variability**		
Kidney disease	42,889	1.12 (1.06, 1.19)
Renal function decline	24,537	1.30 (1.22, 1.38)
ESRD	1,324	1.70 (1.35, 2.14)
**TC to HDL-C ratio variability**		
Kidney disease	42,889	1.08 (1.03, 1.13)
Renal function decline	24,537	1.26 (1.18, 1.33)
ESRD	1,324	1.52 (1.21, 1.89)

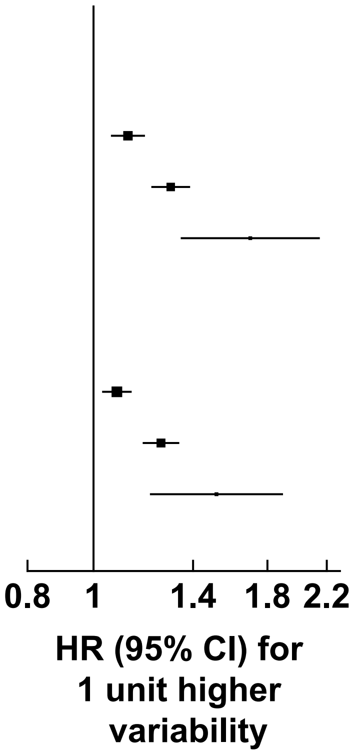

Hazard ratio was adjusted by age, gender, duration of diabetic mellitus, smoking status, body mass index, systolic blood pressure, diastolic blood pressure, haemoglobin A1c, estimated glomerular filtration rate, urine albumin to creatinine ratio, the usages of anti-diabetic drugs, anti-hypertensive drugs, statins and fibrates, Charlson's index and usual LDL-C or TC to HDL-C ratio (as appropriate). LDL-C = Low-density lipoprotein-cholesterol; TC = Total cholesterol; HDL-C = High-density lipoprotein-cholesterol; ESRD = End stage renal disease; HR = Hazard ratio; CI = Confidence interval.

ESM Figure 4c. Hazard ratios for the risk of kidney disease, renal function decline and ESRD with each 1 unit increase in LDL-C (mmol/L) or TC to HDL-C ratio variability using Cox regressions adjusted for baseline covariates in sensitivity analysis 3 (using 36 months collection period for cholesterol)

	Event	
**LDL-C variability**		
Kidney disease	57,030	1.23 (1.17, 1.29)
Renal function decline	33,749	1.41 (1.33, 1.50)
ESRD	2,124	2.27 (1.86, 2.78)
**TC to HDL-C ratio variability**		
Kidney disease	57,030	1.17 (1.12, 1.22)
Renal function decline	33,749	1.37 (1.30, 1.45)
ESRD	2,124	1.84 (1.54, 2.19)

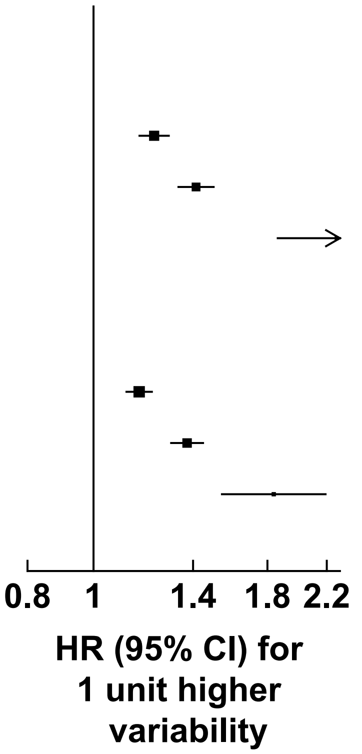

Hazard ratio was adjusted by age, gender, duration of diabetic mellitus, smoking status, body mass index, systolic blood pressure, diastolic blood pressure, haemoglobin A1c, estimated glomerular filtration rate, urine albumin to creatinine ratio, the usages of anti-diabetic drugs, anti-hypertensive drugs, statins and fibrates, Charlson's index and usual LDL-C or TC to HDL-C ratio (as appropriate). LDL-C = Low-density lipoprotein-cholesterol; TC = Total cholesterol; HDL-C = High-density lipoprotein-cholesterol; ESRD = End stage renal disease; HR = Hazard ratio; CI = Confidence interval.

ESM Figure 5. Hazard ratios for the association of a unit increase in LDL−C, TC to HDL−C ratio and triglyceride variability, measured using the coefficient of variation, with kidney disease defined by estimated glomerular filtration rate and kidney disease defined by urine albumin to creatinine ratio from Cox regression models adjusted for baseline covariates

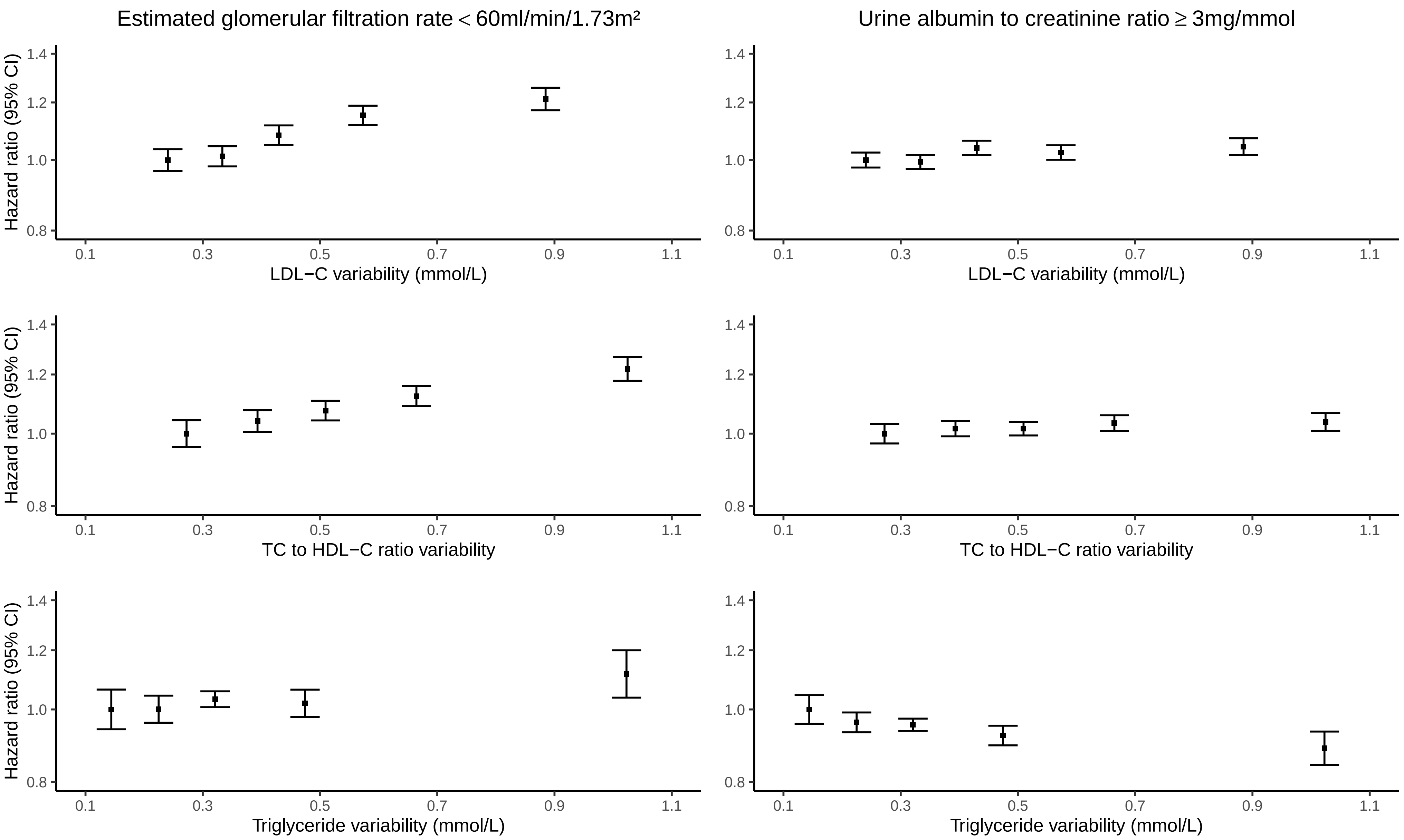

Hazard ratio was adjusted by age, gender, duration of diabetic mellitus, smoking status, body mass index, systolic blood pressure, diastolic blood pressure, haemoglobin A1c, estimated glomerular filtration rate, urine albumin to creatinine ratio, the usages of anti−diabetic drugs, anti−hypertensive drugs, statins and fibrates, Charlson's index and usual LDL−C, TC to HDL−C ratio or triglyceride (as appropriate). CIs are displayed as floating absolute risks. ESRD = End stage renal disease; LDL−C = Low−density lipoprotein−cholesterol; TC = Total cholesterol; HDL−C = High−density lipoprotein−cholesterol.

ESM Figure 6. Hazard ratios for the risk of kidney disease defined by estimated glomerular filtration rate and kidney disease defined by urine albumin to creatinine ratio with each 1 unit increase in LDL-C (mmol/L) or TC to HDL-C ratio variability using Cox regressions adjusted for baseline covariates

	Event	HR (95% CI)
**LDL-C variability**		
Estimated glomerular filtration rate<60ml/min/1.73m^2	20,325	1.44 (1.34, 1.53)
Urine albumin to creatinine ratio≥3mg/mmol	40,458	1.08 (1.03, 1.13)
**TC to HDL-C ratio variability**		
Estimated glomerular filtration rate<60ml/min/1.73m^2	20,325	1.34 (1.25, 1.42)
Urine albumin to creatinine ratio≥3mg/mmol	40,458	1.03 (0.98, 1.08)

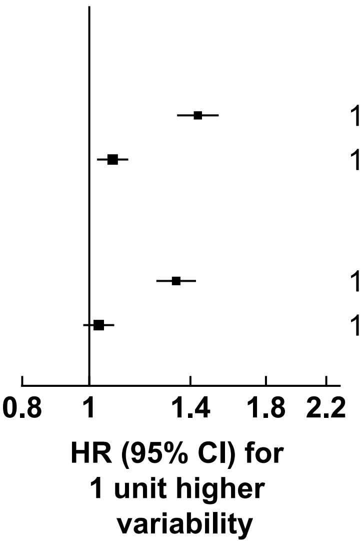

Hazard ratio was adjusted by age, gender, duration of diabetic mellitus, smoking status, body mass index, systolic blood pressure, diastolic blood pressure, haemoglobin A1c, estimated glomerular filtration rate, urine albumin to creatinine ratio, the usages of anti-diabetic drugs, anti-hypertensive drugs, statins and fibrates, Charlson's index and usual LDL-C or TC to HDL-C ratio (as appropriate). LDL-C = Low-density lipoprotein-cholesterol; TC = Total cholesterol; HDL-C = High-density lipoprotein-cholesterol; HR = Hazard ratio; CI = Confidence interval.
